# Pleiotropic effects of recombinant protease inhibitors in plants

**DOI:** 10.3389/fpls.2022.994710

**Published:** 2022-09-02

**Authors:** Phetole Mangena

**Affiliations:** Department of Biodiversity, Faculty of Science and Agriculture, School of Molecular and Life Sciences, University of Limpopo, Polokwane, Limpopo, South Africa

**Keywords:** abiotic stress, biotic stress, genetic engineering, proteolysis, protease enzymes, recombinant protease inhibitors, transgenic lines

## Abstract

Recombinant gene encoded protease inhibitors have been identified as some of the most effective antidigestive molecules to guard against proteolysis of essential proteins and plant attacking proteases from herbivorous pests and pathogenic microorganisms. Protease inhibitors (PIs) can be over expressed in transgenic plants to complement internal host defense systems, *Bt* toxins in genetically modified pest resistance and abiotic stress tolerance achieved through cystatins expression. Although the understanding of the role of proteolytic enzymes and their inhibitors encoded by both endogenous and transgenes expressed in crop plants has significantly advanced, their implication in biological systems still requires further elucidations. This paper, therefore, succinctly reviewed most recently published literature on recombinant proteases inhibitors (RPIs), focusing mainly on their unintended consequences in plants, other living organisms, and the environment. The review discusses major negative and unintended effects of RPIs involving the inhibitors’ non-specificity on protease enzymes, non-target organisms and ubiquitous versatility in their mechanism of inhibition. The paper also discusses some direct and indirect effects of RPIs such as degradation by distinct classes of proteases, reduced functionality due to plant exposure to severe environmental stress and any other potential negative influences exerted on both the host plant as well as the environment. These pleiotropic effects must be decisively monitored to eliminate and prevent any potential adverse effects that transgenic plants carrying recombinant inhibitor genes may have on non-target organisms and biodiversity.

## Introduction

Abiotic stresses such as drought and extreme temperatures, including biotic stress factors like phytopathogenic microorganisms trigger the production of extracellular and intracellular protease enzymes. Proteases, also known as proteolytic enzymes, are a group of digestive enzymes that break down long polypeptide chains into smaller amino acid chains and eventually into single individual amino acids (Ravee et al., 2018). Several studies have indicated that endogenous and exogenous secretion of protease enzymes in plant cell’s cytoplasms is associated with their exposure to biotic and abiotic stress (Morrell and Sadanandom, 2019; Stael et al., 2019; Ali and Baek, 2020; van der Hoorn and Klemencic, 2021). Proteolytic enzymes are responsible for a striking variety of biological processes that include signal initiation, transmission, and termination of many cellular processes (van der Hoorn and Klemencic, 2021). Proteases also play a key regulatory role in plant metabolism by maintaining effective protein quality controls, eliminating nonfunctional proteins, and are used in systemic defense responses. Among these important biological roles, biochemical degradation of cell proteins through hydrolysis of peptide bonds serves as the main primary function of proteolytic enzymes (Morrell and Sadanandom, 2019). However, these enzymes were also found to be associated with the occurrence of cell death (necrosis, excessive chlorosis, and programmed cell death) during senescence of tissues and organs, cell differentiation (Santos and Figueredo, 2021), and additionally acting as critical regulators during embryogenesis, cuticle formation, chloroplast biogenesis and stomatal development (van der Hoorn and Klemencic, 2021). According to D’Ippolito et al. (2021), plant proteases were also found to be involved in signal transductions among phytohormones and the adjustment of stomatal apertures during the exposure of plants to drought stress.

Nevertheless, protease enzymes also induce the formation of reactive oxygen species (ROS) detected during plant exposure and response to abiotic stresses, especially water deficit stress (Liu et al., 2019; Ali and Baek, 2020). Low and feverish temperatures were also reported to induce proteases that diminish plant productivity by causing a rapid burst of ROS in the chloroplasts (Luo and Kim, 2021). The dynamic changes in environmental conditions involving pathogen invasion also caused the expression of distinct digestive enzymes produced either by host plant or invading pathogen and herbivorous insect pests. Al-Ani et al. (2022) demonstrated a trypsin-serine like protease activity of fungi and nematodes during plant parasitism and antibiosis. Most fungal and nematode species inject secretions into plant cells with trypsin-like or serine proteinase activity. For instance, two serine protease enzymes were demonstrated in soybean cyst nematode (*Heterodera* spp. and *Globodera* spp.), and saprophytic fungi used to digest plant tissue proteins to favor the invading pathogenic metabolism and spreading of the infections (Silva et al., 2018; Rodríguez-Sifuentes et al., 2020). Interestingly, plants can produce many different molecules in response to attacks by pathogenic microorganisms and insect pests, or in dealing with the effects of abiotic stresses.

Moreover, the art of recognizing the role of proteolytic enzymes and their inhibitors in plant metabolic systems have led to the development and expression of recombinant protease inhibitors (RPIs). Recombinant protease inhibitors are enhanced protein molecules produced by transgenic plants that are used to inhibit the harmful effects of proteolysis during the exposure of plants to several environmental stress factors. Although, PIs can be naturally expressed by the plants to inhibit the activity of proteases, the RPIs are rather overexpressed by transgenes that are artificially incorporated into host plant genomes using recombinant DNA technology. Several transgenic plants expressing RPIs have been developed and used for resistance against biotic and abiotic stresses, especially for drought tolerance and resistance to plant disease causing pathogens. Some of the major crop species that are genetically engineered to express such recombinant protease inhibitors to reduce proteolytic activities are exemplified in Table 1.

**Table 1:** Recombinant protease inhibitors expressed in transgenic plants and their targeted proteinase enzymes.

Plant species	Common name	Recombinant protease inhibitor	Targeted proteinases	References
*Citrullus lanatus*	Watermelon	Trypsin inhibitor 1	Serine-type endopeptidases	Srikanth and Chen 2016
*Elaeis guineensis*	Oil palm	Mustard trypsin inhibitor	Serine	De Leo et al., 2001
*Glycine max* (L.) Merr.	Soybean	Oryzacystatin I, Oryzacystatin II	Cysteine	Mangena 2020
*Gossypium hirsutum*	Cotton	Potato type I,	Serine	Dunse et al., 2010
*Hordeum vulgare*	Barley			
*Oryza sativa* L.	Rice	Barley trypsin inhibitor, soybean trypsin inhibitors, potato carboxypeptidase inhibitors	Serine	Quilis et al., 2007
*Medicago sativa*	Alfalfa	Oryzacystatin II	Cysteine	Ninkovic et al., 2007
*Saccharum officinarum* L.	Sugarcane	Cysteine protease inhibitor	Cysteine	Soares-Costa et al., 2002
*Solanum lycopersicum*	Tomato	Barley serine protease inhibitor, barley cysteine protease inhibitor	Serine, cysteine	Hamza et al., 2018
*Solanum tuberosum*	Potato	Cowpea trypsin inhibitor, chicken egg white cystatin	Serine, cysteine	Bell et al., 2003), Cowgill et al., 2002
*Triticum aestivum*	Wheat	Potato serine protease inhibitor, potato cysteine protease inhibitor	Serine, cysteine	Gupta et al., 2010, Solomon et al., 1999
*Zea mays* L.	Corn	Barley HvCPI 1-13	Cysteine	Carrillo et al., 2011

Among them, species such as corn, cotton, rice, wheat, and soybean form part of the major crops that are genetically engineered using *Agrobacterium tumefaciens*-mediated genetic transformation to express recombinant genes like *Oryzacystatin I* (*OC-I*), *Oryzacystatin II* (*OC-II*) and cowpea trypsin inhibitor (*Cry1Ac*) for elevated levels of proteinases inhibiting proteins. A handful of studies continue to report the functional significance and efficiency of these partly stable proteins for an increasing number of applications particularly, for the protection of crops against phytopathogens and their potential role as biopesticides (Kim et al., 2009; Grosse-Holz and van der Hoorn, 2016; Clemente et al., 2019). A number of non-target organisms can also be exposed to these RPIs, especially the animals and humans who feed from the RPI containing crops (Im et al., 2021). Recently, the potential risk of *Bt* crops on non-target organisms such as insect pollinators, decomposers, prying insect predators, and the alteration of nutritional value of the crop have drawn a lot of public concerns. The nutritional value of most transgenic plant materials gets limited by the high presence of naturally occurring and induced compounds which interfere with the amounts and quality of nutrients, including nutrient digestion, absorption and assimilation in animals that consume them. Although, in legume crops for instance, postharvest operations such as storage treatments and processing are employed widely for removal of antinutritive factors, potential overexpression of RPIs inherently lowered the quality of food products by enhancing the production of phytic acid (Clarke and Wiseman, 2000). These negative effects necessitated research into breeding for low Bowman-Birk and Kunitz trypsin recombinant protease inhibitors in soybean and other cereal grain crops such as maize, rice and wheat (Rodríguez-Sifuentes et al., 2020). Thus, these and other studies showed that overexpression of RPI genes such as *OC-I*, *OC-II* and *Cry1Ac* exerts a strong influence on crop performance and grain quality.

However, many of these concerns were due to the cultivation of *Bt* crops as reported by Rukarwa et al. (2014) indicating that *Cry* proteins expressed in transgenic sweetpotato had some adverse effects on non-target *Coleopterans* such as ground, rove, and ladybird beetles. In contrast, Yang et al. (2021) reported evidence showing that some protease inhibitors hindered various enzymatic activities in the larval midgut of *Cry* protein resistant *Cnaphalocrocis medinalis*, thereby reducing the insect’s ability to degrade *Bt* toxins. These findings, including many other reports on the pleiotropic effects of transgenic proteins like *Cry* proteins and RPIs are contradictory warranting further research and analysis in the role of these recombinant proteins in the agricultural system. In the current review, potential unintended consequences of recombinant protease inhibitors are discussed, and the gist of postulated direct and indirect impacts of these protease inhibitors on plant health and the environment are also interrogated. A literature survey was limited to biochemical, physiological, and partly morphological pleiotropic effects of RPI overexpression in transgenic plants. But most importantly, the paper deliberates on some of the intrinsic negative characteristics such as ubiquity, non-specificity, and proteolytic degradation of protease inhibitors intended for protection during plant response to biotic and abiotic stresses.

## Stress induced proteolysis in plants

Plants, including many other eukaryotic and prokaryotic organisms comprise a variety of proteins functioning as catalysts, storage, structural, transport and regulatory molecules. Regulatory proteins are those that regulate DNA and RNA expressions as well as cell to cell recognition and signal transductions (Rasheed et al., 2020). Storage proteins, especially those contained within the seeds’ cotyledon comprise essential and/or semi-essential protein molecules serving as building blocks in which their structures and aggregations are key to their functionality in living organisms. Plant seeds contain larger amounts of abundant and usable stored proteins than any other part of the plant, especially when compared to roots and shoots. In leguminous crops 7S and 11S globulins are the most predominant storage proteins, followed by 2S, 9S and 11S globulins (Mouzo et al., 2018). These proteins are synthesized during plant growth and development, accumulating more during seed development within membrane-bound protein bodies and serving as reservoirs of amino acids, reduced sulfur, nitrogen, and carbon molecules required for plant establishment post germination (Dimina et al., 2022).

Other groups of proteins are synthesized based on plants enduring biotic and abiotic stress. Hence, plants exhibiting high sensitivity to environmental stresses such as drought and salinity have also demonstrated higher expressions of protease enzymes under stressful conditions. These proteolytic enzymes are responsible for the catalysis of hydrolytic cleavage of numerous specific peptide bonds, together with the assembly of 2S and 11S globulin storage proteins mostly found in dicot plants (Mangena, 2020). The classification and cleavage of peptide bonds by proteolytic enzymes is based mainly on the catalytic amino acid residue found in the enzyme’s active site (serine protease, cysteine protease, aspartic protease, and metalloprotease). Some molecular and catalytic information of the structure and applications of these protease enzymes are summarized in Table 2. When plants are exposed to stressful conditions, activation of genes that biochemically promote the expression and activity of proteolytic enzymes take place. Even though, proteolysis serves as one of the key catabolic processes in living organisms, protein induction and all metabolic activities that are regulated by these enzymes need to be controlled in order to avoid occurrence of any hazardous actions.

**Table 2:** Classification, general features, and examples of plant-based proteases with their industrial application.

Protease type	Catalytic residue group	Molecular weight (kDa)	Protein	Application
Aspartic protease	Aspartate	30 80	Arctiumisin Cardosin	Alcohol, bioactive peptide production and dairy industry
Cysteine proteases	Cysteine	24.5 28–32.5 23.8 23.4	Actinidin Bromelain Ficin Papain	Fish, animal feed, baking, and textile industry, including bioethanol production.
metalloprotease	Zn^2+^, Ca^2+^ or Mn^2+^	92	MMP-like proteases	Bioactive peptide production and biomedicine
Serine protease	Serine, histidine	55	Carnein Milin	Brewing and dairy industry

Marino and Funk (2011) and, Troncoso et al. (2022).

Total control is necessary because the overexpression of proteolytic enzyme may negatively affect cellular metabolism by hydrolytic degradation of essential proteins. This may take place while plants express proteases for purposes of metabolically counteracting the detriments of stress through dismantling of misfolded and damaged proteins, as well as maintaining sufficient turnover of cellular proteins (Gregersen et al., 2008, 2013; Mahajan and Badgujar, 2010; Diaz-Mendoza et al., 2016). Furthermore, Toderich et al. (2020) also reported a differential composition of essential total proteins and free amino acid content due to salinity stress in seeds of new quinoa genotypes (*Chenopodium quinoa* W.). However, as environmental stresses continue to be the most challenging stress constraints globally, many researchers are thus, prompted to develop transgenic and non-transgenic lines that have been genetically enhanced to increase seed protein yield and oil while circumventing negative effects caused by these growth limiting factors (Taunk et al., 2019; Kumar et al., 2020; Selamat and Nadarajah, 2021).

## Role of protease inhibitors in plants

A major impediment for successful germination, seedling development and overall growth of the plant is the exposure and susceptibility to environmental stress. For years many researchers have been studying the biosynthesis and regulation of specific chemicals associated with defense mechanisms in plants against various stress factors. Some of these chemicals remain unclear while others are considered to be secondary plant metabolites which play key selective regulatory roles during growth, development, and reproduction in plants, and they occur in all other living organisms such as bacteria, fungi, and animals (Demain and Fang, 2000; Isah, 2019). However, the type and concentration of specific chemicals produced by the plant during exposure to stress is determined by various intrinsic and extrinsic factors. These include the plant species/genotype, developmental stage, physiological status, and the environment (Isah, 2019). These factors likewise suggest the adaptive response of plants to stress, defense mechanism and the type of defensive stimuli required.

The specific chemicals used in defense are either constitutive in various plant tissues or are synthesized in response to the exposure to the specific type of stress. On the other hand, complex molecules such as proteins (lectins, enzymes, or enzyme inhibitors), alkaloids and terpenes are inducible constitutive compounds (Moreira et al., 2018). In various plants, proteins that include proteolytic enzymes and protease enzyme inhibitors can be synthesized in response to biotic and abiotic stress. The occurrence of proteases in turn may activate genes that naturally code for the production of protease inhibitors. This system has been widely studied by plant breeding scientists to mostly complement the development of disease and insect pest resistance in transgenic plants. Protease inhibitors constitute approximately 50% of the total amount of proteins found in various crop plants. These proteins include inhibitors of endopeptidases and exopeptidases found under the classification shown in Table 2. Protease inhibitors, therefore, form complexes with these protease enzymes and then inhibit their proteolytic activity, in addition to protecting certain cellular constituents, tissues and fluids (Dunse et al., 2010; Carrillo et al., 2011; Clemente et al., 2019).

Furthermore, some of the protease inhibitors such as potato protease inhibitors (PPI) have a broad spectrum of inhibitory activity. The Kunitz-type serine protease inhibitor serves as the most abundant inhibitor in the Solanaceae and Fabaceae family, representing approximately 44% of the total amount of protease inhibitors in potatoes (*Solanum tuberosum*) with additional 50 and 80% of chymotrypsin and trypsin, respectively (Dunse et al., 2010; Gupta et al., 2010; Herwade et al., 2021). In plant genetic improvement, cysteine and serine protease inhibitors have been widely reported for antidigestive and protection of crops against herbivores (Herwade et al., 2021). Senthilkumar et al. (2009) earlier reported varied inhibiting activity against trypsin and papain proteins, further showing resistance to both insects and phytopathogens. For instance, the report indicated that larvae of *Helicoverpa armigera* that ingested tobacco leaves either died or showed delayed growth and development. Tohidfar and Khosravi (2015) also highlighted the role of cowpea trypsin inhibitor (*CpTI*) which was successfully engineered in several crops (rice, cotton, wheat, rape seed, and eggplant) for protection against attacks by beetles and aphids. *CpTI*, *Bacillus thuringiensis* (*Bt*) and *Bt-*Xtra containing three *CryIAc* from *B. thuringiensis*, *bar* gene from *Streptomyces hygroscopicus* and *pinII* gene from potato coding potato protease inhibitor have also been developed in transgenic plants for abiotic stress resistance (Tohidfar and Khosravi, 2015; Losvik et al., 2018; Lang et al., 2021).

## Pleiotropic effects of recombinant protease inhibitors

Although, numerous studies demonstrated efficient use of RPIs as effective anti-hydrolytic degradation of essential compounds, tissues and for protection of crops against pests and pathogenic organisms. The pleiotropic effects of RPIs in plant protection still need to be clarified. Many scientific and unscientific concerns have been raised in the past, and many more are still emerging due to the fact that the insertion of a transgene into a plant may result to unforeseen and potentially undesirable effects. Roundup Ready (RR) crops serve as excellent example, showing such negative and discouraging pleiotropic effects. Various reports stated that RR crops are responsible for the increasing development of superweeds and other plant types showing resistance to the Roundup (Glyphosate) herbicide. According to Green and Siehl (2021), the sole reliance on glyphosate [N-(phosphonomethyl)glycine, CAS No. 1071-83-6] for weed control potentially led to evolved resistance against this herbicide. In soybean, growth and yield of RR-soy lines were significantly influenced by both the herbicide (Cuvaca et al., 2021) and hot weather conditions causing the splitting of stems due to high lignin content produced (Martens et al., 2018). These unintended effects, however, suggest that increased expression of enzymes or proteins from the transgene may affect the balance of the relevant metabolic pathways. Table 3 summarizes some of the recombinant genes of prokaryotic and eukaryotic origin that are used in the expression of recombinant inhibitors to confer resistance to biotic and abiotic stress in plants. Among them, is the cowpea *CpTI* gene constructed by insertion into a pBIN19 derivative plasmid vector and expressed in plants using *Agrobacterium tumefaciens* through CaMV35S promotor and 3′ NOS terminator (Zhou et al., 2017). These recombinant inhibitors mostly act by either tightly binding to the active site of the protease enzyme as pseudo-substrates or would use trapping, which is a rapid conformational change that traps the cognate protease in a covalent complex fashion (Sabotic and Kos, 2012).

**Table 3:** Recombinant protease inhibitor genes used to engineer plants for biotic and abiotic stress resistance originating from plants, bacteria, and fungi.

Recombinant protease inhibitor gene	Target protein	Inhibitory mechanism	Engineered crop	References
*Alpha-1-antitrypsin*	Trypsin	Tight binding	Tomato	Agarwal et al., 2008
*Serpin*	Cysteine/ papain	Trapping	Rice	Singh et al., 2016
*Aprotinin*	Chymotrypsin	Tight binging	Corn	Sabotic and Kos 2012
*Carboxypeptidase Y inhibitor*	Serine carboxypeptidase Y	Phospholipid binding	Tomato	Abdeen et al., 2005
*CpTI*	Trypsin	Tight binding	Soybean	Clemente et al., 2019
*Pot PI-I*	Proteinase	Tight binding	Cotton	Dunse et al., 2010
*Pot PI-II*	Proteinase	Tight binding	Cotton	Dunse et al., 2010
*Kunitz trypsin-inhibitor-3*	Trypsin	Tight binding	Tobacco	do Amaral et al., 2022
*OC-1*	Cysteine	Tight binding	Soybean	Mangena 2020
*CMe*	Trypsin	Tight binding	Rice	Alfonso-Rubi et al., 2003

### Non-enzyme specificity of protease inhibitors

**Figure 1:**
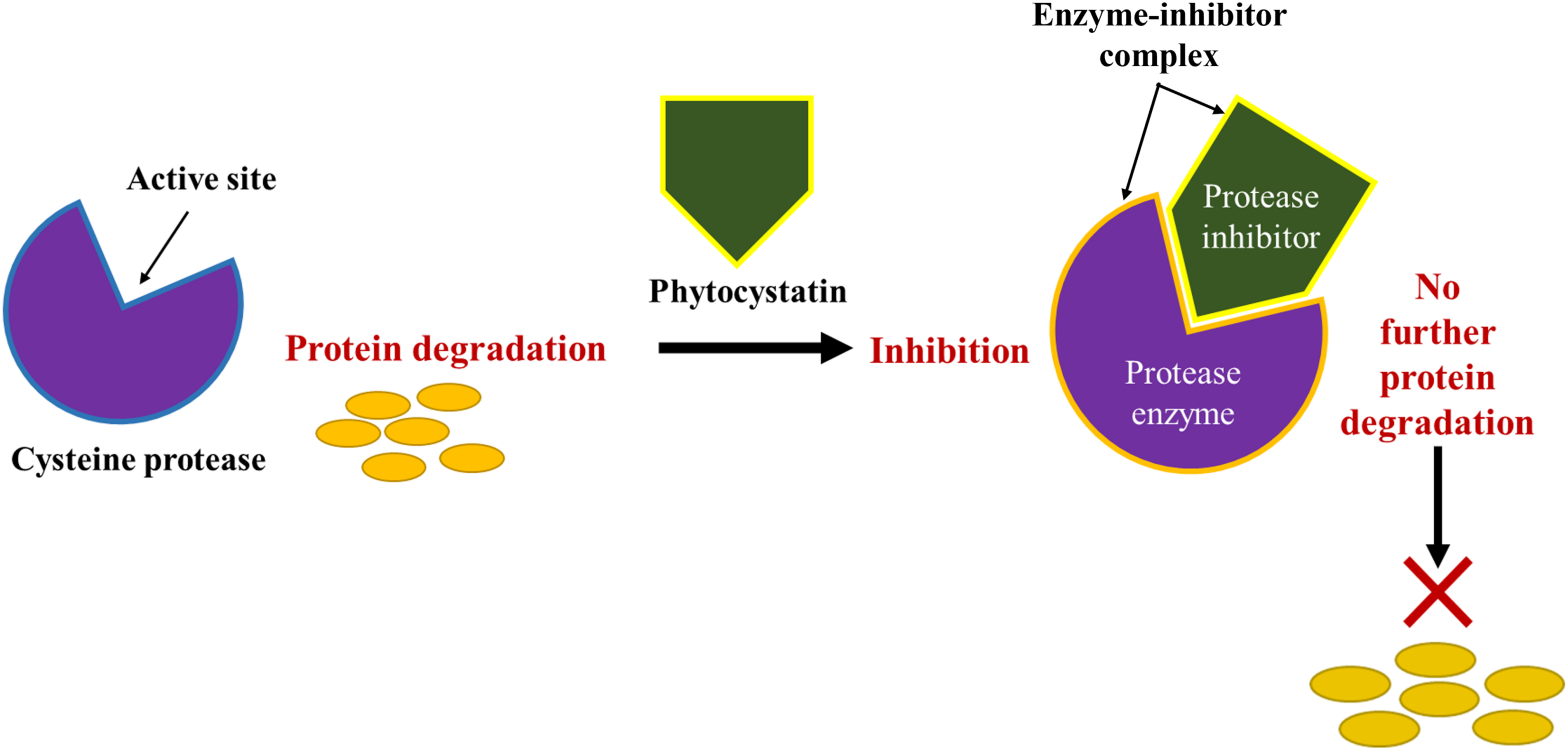
A typical inhibitory action of protease inhibitor (cysteine) on protein degradation through the formation of enzyme-inhibitor complex.

Stress-induced proteolysis also leads to the degradation of proteins into component amino acids residues which ultimately denatures and affect the function of the proteins. It is, however, reported that stress, particularly, abiotic stress causes approximately more than 60% of crop yield losses due to severe changes in protein and secondary metabolite accumulation (Rodziewicz et al., 2019). The composition of cellular proteins is usually altered by environmental conditions, reflecting the true physiological and biochemical outcome of stress on the plant and its genetic capabilities. Depending on the level of stress, plants accumulate or enhance the expression of particular proteins to protect themselves against environmental stress. Classes of proteolytic proteins expressed during stress in plants include endo- and exo-peptidases found within enzyme families of serine proteases, cysteine proteases, aspartic proteases and metalloprotease which were discussed in detail above. Accordingly, these peptidases are able to function individually or as a complex, serving as an active proteolytic machinery (Mangena, 2020). A major problem involving protease is that the proteolytic activity of these enzymes is not limited to the cleavage of a number of bonds or hydrolysis of individual amino acid constituents used as building blocks for the synthesis of new catalytic and structural proteins (Solomon et al., 1999; Gupta et al., 2010; Clemente et al., 2019). However, their activity is also inherently associated with the activation or expression of protein enzymes inhibitors as mentioned in the previous section. These enzyme inhibitors are purposefully expressed to balance and interact in some way with the protease enzyme concentrations to prevent it from causing severe metabolic disruptions leading to tissue senescence. Furthermore, these protease inhibitors also serve a critical role in preventing the progression of pathogenesis resulting from pathogen-induced proteases (Wang et al., 2020). Such enzyme inhibitions could be non-specific within a family of peptidases, affecting the function of proteases in a class having similar mechanisms of action. These inhibitors may cause physical or chemical interactions with enzymes, ultimately and reversibly or irreversibly denaturing the protein portion of the enzyme. Other inhibitors such as the cysteine protease inhibitors inhibit catalytic activity of cysteine proteases by binding to the enzyme’s active site to create a distortion as pseudo-substrate (Table 3). Binding to the active site of the enzyme as indicated in Figure 1 enables the inhibitors to block access of the targeted specific protein substrates for catalysis (Kopitar-Jerala, 2012).

Although, various reports show that increased levels and activity of protease inhibitors were correlated with the plant’s resistance to biotic and abiotic stress, the non-specificity of these protease inhibitors has, however, potently prohibited the growth of plants by significantly altering metabolic processes and interfering with the overall growth and development of plants. Natural protease inhibitors, together with recombinant inhibitors whose concentrations in the cell may be difficult to control can interact with several enzymes in high affinity and even not be easily removed. However, the difficulties expressed by many scientists in developing new and effective RPIs for agricultural or medicinal purposes include problems associated with identifying a specific inhibitor that efficiently blocks the active sites of specific proteolytic enzymes (Figure 1), and the generation of new inhibitors for evolving new targets of proteases. Moreover, other stress types can also be caused or facilitated by multiple protease-mediated processes. Therefore, to continue with the successfully application of RPIs in agriculture, new protease inhibitors need to be discovered and their recombinant genes successfully cloned into bacterial vectors for efficient plant transformation, which remains a challenge and a daunting task. Genetic manipulation of plants also remains highly inefficient, without a routinely successful, genotype independent transformation protocol, and further insights onto the hurdles facing direct or indirect recombinant DNA technology, as well as its genetic, molecular, and regulatory element requirements are thoroughly discussed in a review by Basso et al. (2020). For purposes of efficiently establishing new beneficial and widely functional protease inhibitors such as recombinant serine protease inhibitors (*rBbKI*), serine and cysteine inhibitors (*rBbCI*) derived from native inhibitors discovered from *Bauhinia bauhinioides* seeds, as well as serine and metalloprotease inhibitors from *Enterolobium contortisiliquum* seeds, more knowledge of their structure, selectivity and specificity to the different peptidase enzymes, including their target enzyme activity, must be gathered (Ferreira et al., 2019; Bonturi et al., 2022).

### Ubiquity associated problems

Protease inhibitors are essential tools for maintaining protein balance in the cells to minimize the detrimental effects of biotic and abiotic stress. Evidence of variations in inhibitor levels in response to stress that causes incidences of diverse types of metabolic dysfunctions have also surfaced. Shams and Bano (2017) reported that protease inhibitors like cystatins do not mainly serve as just inhibitors blocking the activity of thiol peptidases, but they also take part in a variety of metabolic and growth processes. In general, protease inhibitors are highly diverse and ubiquitous, inhibiting proteases and other enzymes that are inconstitutive of their natural substrates. Nevertheless, the rejection of transgenic crops by the public and these potential negative effects envisaged from functional diversity of recombinant inhibitors continue to discourage their application in biotechnology. Recombinant PIs’ activity and specificity have been widely emphasized with the purpose of obtaining stress-induced proteins specific inhibitors that are important for accurate and rapid deterrence of particular stress causing factors.

Nonetheless, various reports showed that one of the main limitations to the application of recombinant protease inhibitors containing uniform functionality against more complex stress factors (microbial pathogens and drought) is the fact that environmental stresses can affect the different metabolic pathways at the same time with varied intensity. Furthermore, biotic stresses such as herbivorous pests rapidly evolve and get adapted to the use of a specific RPIs against them, especially, by maintaining diverse digestive enzymes and overexpression of enzymes that are highly insensitive to the recombinant protease inhibitors (Fischer et al., 2015). Meanwhile, overall stability and yield of recombinant protease inhibitors could be achieved by targeting specific enzymes and with protein expression and sequestration taking place in specific cellular compartments.

### Proteolytic degradation of recombinant protease inhibitors

Despite the abundance and diversity of recombinant protease inhibitors found in prokaryotic and eukaryotic cells, significant gene expression barriers in heterologous systems still remains a challenge. Generally, the expression of transgenes encoding RPIs and the transfer, for higher expression of these recombinant protease inhibitor genes in subsequent generations through direct and indirect gene transfer methods such as particle bombardment and *Agrobacterium*-mediated genetic transformation are still problematic. Among the challenges facing gene expression, high level accumulation of recombinant protease inhibitors, improper regulation, and inhibitor proteolysis also presents the most significant barriers to the wider applications of RPIs to confer stress resistance in plants. Some peptidases found in bacteria (*Escherichia coli*) and yeast (*Saccharomyces cerevisiae*) demonstrated a rapid cleavage of recombinant protease inhibitors for purpose of impeding their activity (Gomes et al., 2018; Ma et al., 2020). Similarly, the accumulation of protease inhibitors from transgenic plants’ cytoplasm may lead to the formation of inclusion bodies or be degraded by endogenous proteases. Such effects were also reported by Peng et al. (2019) during the production and recovery of recombinant proteins using biological systems such as bacteria and yeasts for pharmaceutical and medicinal purposes.

In simpler terms, the expression or accumulation of recombinant protease inhibitors in transgenic plants may be recognized as “foreign or abnormal” protein bodies triggering their rapid degradation through various well-characterized ubiquitin-mediated proteolytic pathways. Although not many reports present the mechanism of interaction between intracellular proteases and recombinant proteases in plants, the degradation pathways appear to be similar to those in bacterial and fungal cells. Viegas et al. (2017) presented evidence indicating that some leaf vacuolar proteases active under mildly acidic pH significantly altered the efficiency and integrity of recombinant protease inhibitor proteins. This study also emphasized the fact that, specific mechanisms underlying the action of these plant proteins against recombinant proteins remains unknown. However, in contrast with microbial production of recombinant protease inhibitors for pharmaceutical purposes and other industrial applications, plant proteases taking part in proteolysis of recombinant proteins, and mutant plants that lack proteases potentially damaging to RPIs are not available for crop improvement purposes. Therefore, according to Viegas et al. (2017), Jutras et al. (2019), and others, future research should focus on devising specific strategies for counteracting the effects of vacuolar proteases by identifying and characterizing their specific proteolytic activities in plants. However, for purposes of recombinant protein extraction in plant tissue for industrial uses instead of conferring stress tolerance, these proteins can be accumulated in extracellular compartments and in the endoplasmic reticulum (ER) *via* secretory pathways to prevent and control proteolysis in transgenic plant cells (Viegas et al., 2017; Gomes et al., 2018; Jutras et al., 2019; Peng et al., 2019).

### Phenotype overexpression

Interestingly, several recombinant protease inhibitor genes are regulated in stressed plants. Gene products like RPIs have been widely identified and characterized in detail for their antinsecticidal, antimicrobial and antiviral properties, especially with artificial feeding experiments involving different transgenic lines. Dang and van Damme (2015) reported transgenic plants encoding pokeweed antiviral protein (*PAP*), curcin 2 and dianthin from *Phytolacca americana*, *Jatropha curcas* and *Dianthus caryophyllus*, respectively. The transgenic lines exhibited increased resistance to *Rhizoctonia solani* Kuhn, a soil-inhibiting parasitic fungi that causes collar rot, root rot, damping off and wire stem disease in cultivated crop plants (Butler, 2018). The *PAP* gene was introduced into plant species such as tobacco and potato plants by genetic transformation using *A. tumefaciens*. All transgenic plant expressing *PAP* or mutant derivative of the *PAP* gene showed enhanced resistance to different viral infections. However, overexpression of recombinant proteins remains a complex biological process that is not well understood, may lead to herbicide/pesticide resistance and disruption of the overall growth processes in plants (Losvik et al., 2018; Clemente et al., 2019). In most cases, overexpression of RPIs have led to improved stress resistant phenotypes. Losvik et al. (2018) reported upregulation and overexpression of a recombinant protease inhibitor, *C12c* controlling resistance against aphids in barley (*Hordeum vulgare* L.).

Overexpression of *Brassica oleracea* cysteine protease inhibitor (*BoCP1*) was also reported to reduced total protease activity while retaining cellular soluble protein content and delaying postharvest senescence by down-regulating different senescence-regulating cysteine protease genes (Tan et al., 2017). *Malus prunifolia* cystatin 4 (*MpCYS4*) localized in the nucleus, cytoplasm and plasma membrane of onion epidermal cells (Tan et al., 2017) resulted in ABA-hypersensitivity. Nevertheless, it should be noted that, although such enhanced ABA-induced stomatal closures and altered expression of ABA-induced stress responsive genes improved drought stress tolerance, prolonged closure of stomata may negatively influence plant growth, development and recovering of plants to stress. Under normal circumstance, ABA sensitivity facilitate shoot growth and root development, enhancing salt and drought stress tolerance in transgenic plants (Sun et al., 2020). Limited effects on plant stress avoidance were also reported by Plessis et al. (2011) and Bi et al. (2019) due to the expression of ABA-hypersensitivity in mutant plants.

### Biochemical and physiological effects

As briefly described on the previous section, the overexpression of recombinant protease inhibitors triggers ABA-hypersensitivity which signaled prolonged closure of stomata in *Arabidopsis* (Bi et al., 2019). Inevitably, the closure of stomata was brought about by the reduction in turgor pressure following a massive efflux of potassium ions (K^+^) and anions from guard cells, inhibiting the activity of plasma membrane H^+^-ATPase and CO_2_ uptake for photosynthesis. Reduction in the negative impacts of drought and other abiotic stresses is associated with increased water use efficiency (WEU) occurring under lower physiological control of stomatal conductance (Haworth et al., 2016). Most protease inhibitors in plants are proteinaceous competitive inhibitors that tightly bind to the active sites of proteases to cause detrimental disruption of processes catalyzed by these enzymatic proteins. However, the overexpression of RPIs can potentially inhibit the role of small ubiquitin-like modifier (SUMO) proteins regulating the normal functioning of metabolism during exposure to biotic and abiotic stress. In plants, SUMO mediated cellular processes are induced by heat, drought, and oxidative stress whereby these proteins are involved in maintaining genome stability, chromatin regulation, transcription, translational RNA splicing, ribosome biogenesis and other cell cycle-related processes (Morrell and Sadanandom, 2019).

Many SUMOylation proteins (15–20%) playing critical roles ranging from proteasomal degradation, biosynthesis of complex macromoles and regulation of individual protein activities during stress could be inhibited by RPIs overexpressed in transgenic plants. Furthermore, Hou et al. (2018) indicated that all SUMO proteases are cysteine proteolytic enzymes which can be easily and rapidly inhibited by cysteine protease inhibitors. Cysteine proteases are specialized proteases found widely in all eukaryotic organisms, including transgenic and non-transgenic plants, and play a key role in many growth processes ranging from germination to plant tissue senescence (Morrell and Sadanandom, 2019). Above reports generally indicate the metabolic or physio-biochemical interference effects caused by protease inhibitors which will be better explained by the unintended environmental effects noted for *Bt* toxins and other non-target organisms discussed in the next topic. Additionally, protease inhibitors also exhibit direct interfering effects on endogenous proteases altering the physiological or compositional characteristics of the transgenic host plant. In this case, RPIs could rapidly interfere with the regulation of several metabolic processes such as the elimination of misfolded proteins, polypeptide pre- and pros-region processing during protein maturation and turnover of certain essential proteins (Solomon et al., 1999; Buono et al., 2019; Bonturi et al., 2022).

### Environmental effects

In humans for instance, the occurrence of several inherited disease such as epilepsy and emphysema have been attributed to the pleiotropic effects of some specific protease inhibitors (Clemente et al., 2019). Various reports suggested significant effects of RPIs on negligible phenotypic changes, metabolic changes, insensitivity to protease inhibitors and inhibition of non-targeted organisms and proteins. Clemente et al. (2019) also discussed the potential role of serine protease inhibitors for herbivorous insect control which indiscriminately affect insect larvae of non-target organism. Serine protease inhibitors expressed in transgenic plant tissues were mobilized into the insect digestive tract along with the food and then blocked protein digestion leading to insect malnutrition and eventually its growth and development retardation. Serine protease inhibitors such as soybean Kunitz and Bowman-Birk inhibitors have been characterized for their potential control of herbivores. But major limitation arose when the use of these overexpressed recombinant proteins prohibited the utilization of transgenic plants for food and feed manufacturing as they also serve as antinutritional factors (Mittal et al., 2021).

Furthermore, serine-type inhibitors bovine aprotinin and tomato Kunitz-type cathepsin D inhibitor expressed in potato caused altered leaf protein contents expressed ectopically in transgenic crop plants (Munger et al., 2012). Several latest studies still describe the use of recombinant protease inhibitors as potent pesticides (Shams and Bano, 2017; Bonturi et al., 2022); however, these herbivorous insects also developed various strategies to cope with the dietary protease inhibitors. These evolutionary strategies render the use of recombinant protease inhibitors ineffective as evolving pests also demonstrate the ability to overexpress proteolytic enzymes to outcompete inhibitory proteins, and likewise use alternative classes of proteases to improve their insensitivity against the inhibitors. The inhibitory potency of RPIs against insect pests and pathogenic infection continues to be investigated since many researchers believe that the benefits outweigh their disadvantages. Other unintended effects of RPIs include their inhibitory role against proteases in non-targeted organisms which recently also caused a serious public uproar. Arpaia et al. (2021) reported a decline in bee pollinator populations in Europe as a result of both natural and entropic environmental factors. Nevertheless, traits such as *Cry* gene-based toxins and double strand RNA (dsRNA) are implicated on having lethal and sublethal effects on non-target species such as the insect pollinators. According to literature, recombinant protease inhibitors may directly or indirectly affect non-target organisms through the establishment of formal ecological interactions and through intermediary herbivorous/carnivorous feeding among organisms with the one that primarily fed on the recombinant material (Abbas, 2018; Lang et al., 2019; Dang et al., 2021).

## Final considerations and conclusion

A survey of current scientific literature indicates that proteolytic enzymes and their inhibitors play a crucial role in various biological processes involving the degradation of essential metabolic proteins, regulation of cellular protein catabolism and the inhibition of proteases induced during the exposure of plants to environmental stress. Most living organisms, including plants mainly contain serine proteases, cysteine proteases, aspartic proteases and some metalloproteases that are primarily involved in protein digestion and detoxification (Yang et al., 2021). When combined with environmental stress, proteolytic enzymes could be very debilitating to crops and plant life in general. Recombinant protease inhibitors have been expressed in various crop species to specifically confer resistance and protection against such various types of stress factors. RPIs play the most important function of keeping endogenous proteases’ digestive activities under control, while preventing invasion and attacks by pathogenic microorganisms and insect pests.

Recombinant genes overexpressing these RPIs were then introgressed in many horticultural crops for the aforesaid reasons. Nevertheless, these RPIs have been implicated in harming a number of non-target organisms (Rukarwa et al., 2014). The most obvious were pollinators interacting with flowers of transgenic plants, and predators that feed on targeted insect pests that has consumed plant materials containing RPIs. All non-target organisms will be severely affected, especially if they fail to express enzymes that could digest the inhibitors to detoxify the protein. So far, reports show that these organisms are mainly affected by PIs found in transgenic materials either directly or indirectly. Inadvertently, potential phenotypic changes in the transgenic plants as a result of transgene expression may directly affect pollinators or pollination patterns. Other pleiotropic effects arising from altered biochemical pathways with changes in essential metabolic products, abundance of unwanted byproducts (ROS), expression of new types of proteases and several phenotypic consequences may also occur.

Consequently, both proteases and their inhibitors may be harmful to pests, despite being essential for the maintenance and survival of plants during their acclimation to stressful habitat conditions, causing phenotypic and cellular disruptions when present in the cells in higher concentrations (Sharma and Gayen, 2021). However, unlike many other studies in genetically modified crops, the application of RPIs for plant improvement did not cause a spike in studies evaluating their negative impacts on living organisms and environment. Thus, pleiotropic effects of RPIs are not easy to assess once transgenic lines are not associated with major apparent environmental risks but are related more with cost reductions of adopting the technology for improved crop performance and productivity, both in the field and during postharvest processing. But, going beyond these effects, application of RPIs is still encouraged without limitations since they contribute to cost and risk reductions, especially in the pharmaceutical industry, and further contribute to reduced risk associated with the use of chemical pesticides. A major impediment to increasing crop yield is that the exposure of plants to environmental stress is frequently coupled with the expression of proteolytic enzymes. Nonetheless, the overexpression of proteases in plant cells may differ according to the type and level of stress, and the plant genotype-dependent resistance. In the initial stage during metabolism, protease enzyme expression often serves as mediators of signal initiation during the onset of stress, escalations occur leading to the termination of certain cellular processes and then followed by hormonal inductions as the stress progresses (Ali and Baek, 2020; D’Ippolito et al., 2021). All of these effects emphasize the need for scientists to continue research in recombinant protease inhibitor expression for regulating protease activity, but modulation should be accompanied by very minimal pleiotropic effects on crop’s life cycle, animal and human health, and the environment.

## Author contributions

The author confirms being the sole contributor of this work and has approved it for publication.

## Conflict of interest

The author declares that the paper was prepared in the absence of any commercial or financial relationships that could be construed as a potential conflict of interest.

## Publisher’s note

All claims expressed in this article are solely those of the authors and do not necessarily represent those of their affiliated organizations, or those of the publisher, the editors and the reviewers. Any product that may be evaluated in this article, or claim that may be made by its manufacturer, is not guaranteed or endorsed by the publisher.

## Abbreviations

*Bt*: *Bacillus thuringiensis*
Cry: Crystal
*OC-I*: *Oryzacystatin I*
*OC-II*: *Oryzacystatin-II*
PI: Protease inhibitor
ROS: Reactive oxygen species
RPI: Recombinant protease inhibitor
